# Determination of Construction Temperatures of Crumb Rubber Modified Bitumen Mixture Based on CRMB Mastic

**DOI:** 10.3390/ma12233851

**Published:** 2019-11-22

**Authors:** Yanan Li, Yuchao Lyu, Meng Xu, Liang Fan, Yuzhen Zhang

**Affiliations:** 1 College of Chemical Engineering, China University of Petroleum, Qingdao 266580, Shandong, China; liyanan2992@163.com (Y.L.); B15030098@s.upc.edu.cn (Y.L.); xumeng04@126.com (M.X.); 2 Shandong Transportation Research Institute, Jinan 250000, Shandong, China

**Keywords:** crumb rubber modified bitumen mastic, Brookfield viscosity, mixing and compaction temperatures

## Abstract

Crumb rubber modified bitumen (CRMB) has been widely used in pavement construction and provides an effective way to recycle waste tires and helps alleviate the “black pollution” problem. There are no current specifications regarding the appropriate mixing and compaction temperatures of the CRMB mixture. There is a direct relationship between the mixing and the compaction temperatures of the CRMB mixture and the viscosity of the CRMB mastic. In this study, we first prepared CRMB using crumb rubber powder and penetration grade 70 neat bitumen, then prepared the CRMB mastic using CRMB and fillers (limestone mineral powder and cement). Finally, we used the CRMB mastic and aggregate to make mixture specimens. The best air void of the specimens was subsequently used to demarcate the viscosity of the CRMB mastic, and the construction temperatures (including the mixing temperature and the compaction temperature) were calculated based on the viscosity of the CRMB mastic from the viscosity–temperature curves. Test results indicated that the best viscosity of the CRMB mastic was 2.7 ± 0.2 Pa·s and 3.9 ± 0.3 Pa·s that corresponded to the mixing and compaction temperatures, respectively.

## 1. Introduction

Increasing attention has been paid to modified bitumen and modified bitumen mixture over time. Pavement materials require a higher quantity of bitumen mastic because of the higher volume of traffic, load, and dynamic water pressure they are exposed to. Rubber powder made from old tires is added to neat bitumen to obtain modified bitumen, which is termed crumb rubber modified bitumen (CRMB) or rubber bitumen. The CRMB mixture has a favorable ageing resistance, fatigue restraint ability, high-temperature stability, low-temperature anti-cracking performance and water resisting properties. It also contributes to alleviating the “black pollution” problem. Stone mastic asphalt (SMA) displays good pavement performance and has been widely applied in pavement construction. Bitumen mastic plays a key role in SMA; however, there is increasing concern about the use of it. Improved adhesive attraction between the bitumen and aggregate occurs only after the formation of the bitumen mastic by bitumen absorbing onto the surface of the filler. Bitumen mastic is one of the three space grid structure dispersed phases in modern mastic theory, being the first and most important phase. The filler is the dispersed phase and the bitumen is the medium. The structure, composition and performance of the bitumen mastic have a direct bearing on the performance of the pavement composed of the bitumen mixture [1]. Therefore, research only on the bitumen binder is inadequate and it is more important to research the bitumen mastic. However, most road workers ignore the dispersed effect of the filler and the interface chemistry effect between the filler and the bitumen and just treat the filler as an inert filler. In addition, although the bitumen binder itself is good, the actual pavement performance is less than satisfactory. Therefore, it is necessary to study the material made from the filler and the bitumen, i.e., the bitumen mastic.

Puzinauskas, V. found that the filler has two functions in the bitumen mixture when he researched cement–filler bitumen mixtures [2]. He found that the filler was not just a pure filler, but was combined with the bitumen to obtain the bitumen mastic. Tunnicliff, D.G. found that mineral powder below 0.075 mm had a great effect on the water stability and the anti-aging performance of bitumen mixtures [3]. Huang Baoshan et al. chose three filler types and four different filler–bitumen ratios to study bitumen mastics, and the results showed that the filler–bitumen ratio must be controlled by the overall performance of the bitumen mixture [4]. Wu Yuhui used a dynamic shear rheometer (DSR) and a bending beam rheometer (BBR) to study the effect of mineral powder content on the high- and low-temperature performance of penetration grade 90 neat bitumen mastic [5]. The results showed that a filler–bitumen ratio between 0.8 and 1.2 could provide a balance between high- and low-temperature performance.

Previous studies showed that the construction temperatures of the CRMB mixture are significantly higher than that of the road bitumen mixture due to the higher viscosity of the CRMB, which leads to higher levels of energy consumption and air pollution [6,7,8,9,10]. There are no specifications for determining the construction temperatures of the CRMB mixture. Several researchers have concentrated on studying the construction temperatures of modified bitumen in recent decades. Yildirim, Y. et al. used high shear rate viscosity–temperature curves to determine the construction temperatures of modified bitumen mixture [11]. In 2001, Bahia, H.U. et al. used the change in zero shear viscosity to determine the construction temperatures by changing the shear rate [12]. In 2003, Reinke, G. obtained the construction temperatures by the shear stability viscosity of DSR [13]. Akisetty, C.K. et al. used the phase angle and the angular frequency of DSR to determine the construction temperatures [14]. West, R.C. et al. [15] and Cai Jun et al. [16] used the air void to determine the construction temperatures of warm mix rubber bitumen mixtures. Ozturk, H. I. et al. found that the compaction temperature could be lowered by 15 °C and warm mix asphalt additive can be used as a compaction aid to lower the compaction effort [17].

The research regarding bitumen mastic to date has been concentrated on mostly non-modified bitumen mastic. There is limited research on the construction temperatures of the CRMB mixture, if any, based on the air void of the CRMB mixture. Hence, studying the determination of the construction temperatures of the CRMB mixture based on the viscosity of the CRMB mastic is required. In the present study, the viscosity of the CRMB mastic was used to determine the construction temperatures of the CRMB mixture, and the temperatures were verified by the air void of the CRMB mixture.

## 2. Materials and Methods

### 2.1. Raw Materials

Penetration grade 70 neat bitumen produced in SsangYong, Incheon, Korea, was used in the present study. Table 1 summarizes the properties of this bitumen binder based on the Chinese standard JTG E20-2011 [18] and the test method by the Strategic Highway Research Program A [19].

The rubber powder (20 mesh size) was made from truck tires in Changzhou, China. Table 2 and Table 3 summarize the physical properties and the chemical properties of the rubber powder, respectively.

The fillers were limestone mineral powder and cement (strength degree of 42.5). Table 4 summarizes their various properties.

The diameter of the mineral powder and cement used in the present study was smaller than 0.075 mm. Table 5 summarizes the screening results of the mineral powder.

The aggregate used in the CRMB was basalt. Table 6 and Table 7 summarize the technology parameters of the basalt.

### 2.2. Preparation of Bitumen Mastic

The present study used CRMB binder and CRMB mastic to compare the accuracy of determination based on viscosity. The CRMB mastic was made by adding the filler to the CRMB binder. Hence, the preparation process parameters did not influence the comparison results and the parameters have been commonly used.

The CRMB binder was made by adding rubber powder (20 mesh size) to penetration grade 70 neat bitumen. The mass ratio of rubber powder to penetration grade 70 neat bitumen was defined as the generalized filler–bitumen ratio. The ratios used were 0.16, 0.18, 0.20, and 0.22. The preparation parameters of the generalized base bitumen mastic were as follows: the mixing temperature was 180 °C, the mixing time was 45 min, and the shear rate was 1000 r/min.

**Figure 1:**
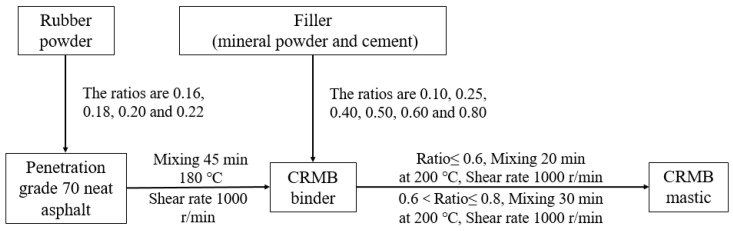
Preparation processes of the crumb rubber modified bitumen (CRMB) binder and the CRMB mastic.

The CRMB mastic was made by adding fillers to the CRMB binder. The ratio of filler to CRMB binder was defined as the filler–bitumen ratio. The ratio of filler was between the weight of the mineral powder and the rubber bitumen. The ratios used were 0.10, 0.25, 0.40, 0.50, 0.60, and 0.80. We used a small mixer in the laboratory to mix the samples by mechanical agitation. The preparation parameters of the rubber bitumen were as follows: the mixing temperature was 180 °C, the mixing time was 45 min, the shear rate was 1000 r/min and they were the same for different ratios of samples. The preparation parameters of the CRMB mastic were as follows: the mixing temperature was 200 °C, the mixing time was 20 min when the ratio was less than 0.6 and was 30 min when the ratio was less than 0.8, and the shear rate was 1000 r/min. The preparation processes for the CRMB binder and the CRMB mastic are shown in Figure 1.

### 2.3. Research Methodology

There are three commonly used methods for determining the viscosity of bitumen. The present study used the Brookfield viscosity method, which tested the viscosity of the bitumen binder and the bitumen mastic at different temperatures. Then, the construction temperatures were calculated by the relationship between the viscosity and temperature. Table 8 summarizes the test parameters for the CRMB that is used mainly in America [20].

## 3. Results and Discussion

### 3.1. Determination Based on the CRMB Binder

Four groups of the CRMB binder were tested based on the different ratios described previously and the viscosity–temperature curves were calculated with the Saal formula (Equation (1)) from the ASTM D2493 standard:

Lglg (η × 1000) = n − m × lg( T + 273.15)(1)

where, η is the viscosity (Pa·s) and T is the temperature (°C).

**Figure 2:**
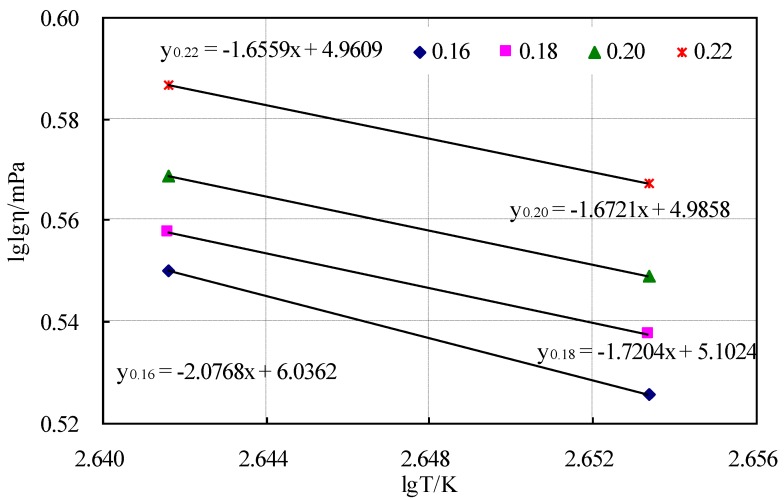
Viscosity–temperature curves of the CRMB binder.

The viscosity–temperature curves calculated by Equation (1) using the viscosity of the CRMB binder at 165 °C and 177 °C are shown in Figure 2.

The viscosity ranges of the mixing and the compaction temperatures were 0.17 ± 0.02 Pa·s and 0.28 ± 0.03 Pa·s, respectively, based on the Chinese standard JTG E20-2011 [18]. The construction temperatures were obtained from Figure 2 and are shown in Table 9.

Table 9 shows that the construction temperatures were too high and against real-life engineering. Thus, these construction temperatures that were determined based on the viscosity of the CRMB binder are inaccurate.

### 3.2. Determination Based on the CRMB Mastic

#### 3.2.1. The Relationship between Viscosity and Temperature of the CRMB Mastic

**Figure 3:**
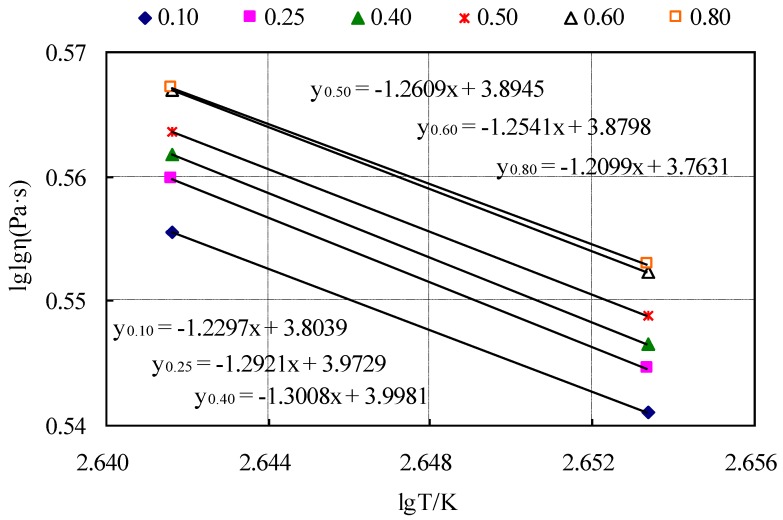
Viscosity–temperature curves of the CRMB mastic.

Six groups of the CRMB mastic were tested based on the different ratios described previously and the viscosity–temperature curves were calculated with Equation (1) from the ASTM D2493 standard. Figure 3 shows the viscosity–temperature curves made with the Brookfield viscosity method at 165 °C and 177 °C.

#### 3.2.2. Demarcating the Viscosity Ranges of the CRMB Mastic

The air void is a very important index of the bitumen mixture. The type of CRMB mixture was AR-AC-13. The generalized filler–bitumen ratio was 0.18. The shaping method used was the superpave gyratory compactor (SGC). The best air voids of the CRMB mixture were used to calculate the viscosity ranges of the construction temperatures. The other construction temperatures of the mixture at the different generalized filler–bitumen ratios were calculated using the viscosity ranges.

Table 10 shows three types of gradation (A, B and C) of the CRMB mixture. The bitumen aggregate ratio was 8.2%.

The air voids of three types of gradation are shown in Table 11.

**Figure 4:**
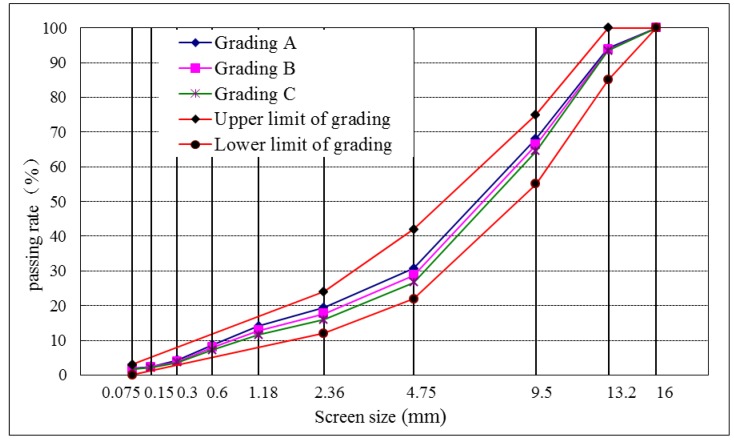
Gradation curves of the CRMB mixture.

Table 11 shows that the air voids of gradation B and gradation C were satisfactory. The present study chose gradation C as the experimental gradation next, as shown in Figure 4, and Table 12 summarizes the volume index of the CRMB mixture.

**Figure 5:**
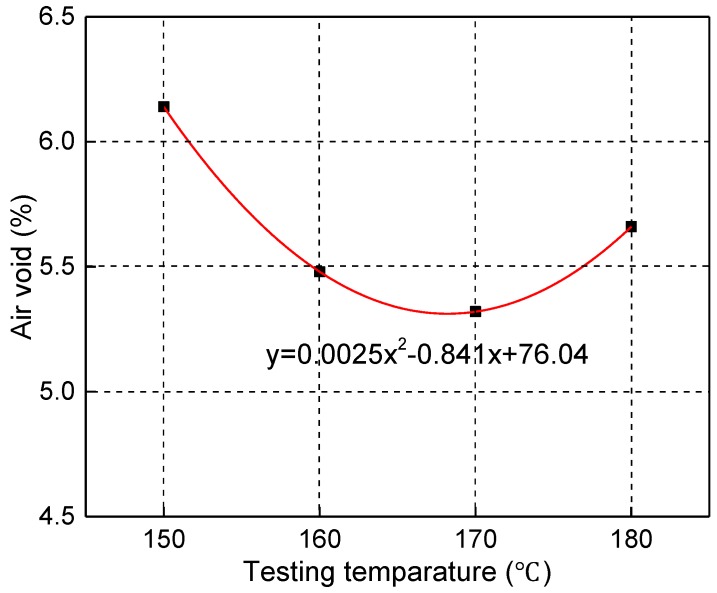
Relationship between the air voids and the compaction temperatures.

The mixing temperature was approximately 170–180 °C for the actual construction. Therefore, the mixing temperature was approximately 170–180 °C in the test and the compaction temperatures were 180 °C, 170 °C, 160 °C, and 150 °C using the SGC. The results are shown in Figure 5.

Figure 5 shows that the relationship between the air voids and the compaction temperatures was a hyperbolic curve. It can be seen that with increasing the compaction temperature, the air voids first decreased, reaching a minimum level, and then began to increase gradually. Furthermore, it can be calculated from the above figure that the CRMB mixture has the lowest air void when the compaction temperature is 168.2 °C. Therefore, a compaction temperature range of 164–172 °C was determined. Generally, the mixing temperature is higher than the compaction temperature by approximately 10 °C [21,22,23,24]. Therefore, a mixing temperature range of 174–182 °C was determined. Corresponding to the viscosity–temperature curve y0.18 = −1.7204x + 5.1024 in Figure 2, the viscosity ranges of the mixing and compaction temperatures were selected as 2.4–3.1 Pa·s and 3.3–4.5 Pa·s, respectively. In order to avoid the range of construction temperatures being too wide, the final viscosities for mixing and compaction were selected as 2.7 ± 0.2 Pa·s and 3.9 ± 0.3 Pa·s, respectively.

#### 3.2.3. Determination of Construction Temperatures Using Viscosity of the CRMB Mastic

Corresponding to the viscosity–temperature curves in Figure 3, the mixing and compaction temperatures were calculated by the viscosity of the CRMB mastic and the results are shown in Table 13.

Table 13 shows that the difference between the mixing and the compaction temperatures was about 12–17 °C, which agrees with engineering practice overall. However, the difference between the mixing and the compaction temperatures was not approximately 10 °C, with the reason for this requiring further study.

## 4. Conclusions

The viscosity ranges of the mixture binder at the mixing and compaction temperatures were 0.17 ± 0.02 Pa·s and 0.28 ± 0.03 Pa·s based on the Chinese standard JTG E20-2011, respectively. However, it can be seen from the data in this paper that the construction temperatures calculated based on the viscosity of the CRMB binder were too high and were inconsistent with the actual construction parameters, that is, the construction temperatures of the CRMB mixture determined by this method may be inaccurate. The results of this paper show that the construction temperatures of the CRMB mixture can be determined based on the viscosity of the CRMB mastic instead of the CRMB binder and the construction temperatures determined according to this recommendation are consistent and feasible with engineering practice overall. In detail, it is recommended that the viscosity ranges of the CRMB mastic in the mixing and compaction process should be 2.7 ± 0.2 Pa·s and 3.9 ± 0.3 Pa·s, respectively.

## Figures and Tables

**Table 1:** Properties of the penetration grade 70 neat bitumen used in the present study.

Properties		Requirements	Test Results
Penetration (25 °C; 0.1 mm)		60–80	68.9
Ductility (15 °C; cm)		≥40	68
Softening point (°C)		≥46	48.3
Flash point (°C)		≥230	263
Wax content (%)		≤3	1.84
Density (g/cm^3^)		report	1.032
Solubility (trichloroethylene; %)		≥99.5	99.7
RTFOT (163 °C, 75 min)	Mass change (%)	≤0.8	0.3
	Retained penetration (%)	≥58	78
	Ductility (15 °C; cm)	≥15	44
PG grade		64–22	

**Table 2:** Physical properties of the rubber powder.

Testing Items	Relative Densities	Water Ratio (%)	Metal Content (%)	Fiber Content (%)	Screenings (%)
Test results	1.16	0.6	0.01	0.5	8.3
Requirements	1.10–1.30	<1	<0.05	<1	<10

**Table 3:** Chemical properties of the rubber powder.

Testing Items	Ash Content (%)	Acetone Extract (%)	Carbon Black Content (%)	Rubber Hydrocarbon Content (%)
Test results	7.2	4.9	31.3	55.2
Requirements	≤8	≤22	≥28	≥42

**Table 4:** Properties of the mineral powder and the cement.

Kinds of Filling	Apparent Density (g/cm^3^)	Hydrophilic Coefficient	<0.075 mm Content (%)
Mineral powder	2.71	0.89 (<1)	90.4
Cement	2.80	0.78 (<1)	92.3

**Table 5:** Sieve analysis of the mineral powder.

Screen Size (mm)	Passing percent (%)
0.075	83.4
0.15	91.2
0.3	97.3
0.6	100

**Table 6:** Test results of the aggregates’ properties.

Aggregate Properties	#1 (9.5–16 mm)	#2 (4.75–9.5 mm)	#3 (2.36–4.75 mm)	#4 (0–2.36 mm)
Apparent density (g/cm^3^)	2.868	2.857	2.839	2.865
Bulk density (g/cm^3^)	2.797	2.766	2.757	2.749
Water absorption (%)	0.89	1.15	1.05	1.47

**Table 7:** Analysis results of different mineral aggregates.

Screen Size (mm)		16	13.2	9.5	4.75	2.36	1.18	0.6	0.3	0.15	0.075
Passing-percent (%)	#1	100	83.2	10.4	0.3	0.3	0.3	0.3	0.3	0.3	0.3
	#2	100	100	98.5	12.1	0.6	0.6	0.6	0.6	0.6	0.6
	#3	100	100	100	85.3	25.7	14.0	8.1	4.0	2.9	1.9
	#4	100	100	100	100	86.3	64.1	39.2	18.5	10	7.6

**Table 8:** Test parameters of the CRMB binder and the CRMB mastic.

Test Parameters	Rotor	Revolving Speed (r/min)
CRMB binder	27#	20
CRMB mastic	27#	30

**Table 9:** Construction temperatures of the CRMB mixture.

Generalized Filler–Bitumen Ratio	0.16	0.18	0.20	0.22
Mixing temperature (°C)	269–281	299–315	313–329	329–346
Compaction temperature (°C)	246–256	270–283	282–295	298–311

**Table 10:** Three grading results of the CRMB mixture.

Types of Gradation #1:#2:#3:#4	Screen Size (mm)									
	16.0	13.2	9.5	4.75	2.36	1.18	0.6	0.3	0.15	0.075
A (35:38:7:20)	100.0	94.1	68.1	30.7	19.4	14.1	8.7	4.3	2.5	2.0
B (37:38:7:18)	100.0	93.8	66.3	28.7	17.7	12.9	8.0	3.9	2.3	1.8
C (39:38:7:16)	100.0	93.4	64.5	26.7	16.0	11.6	7.2	3.6	2.1	1.7

**Table 11:** Volume analysis of preliminary grading.

Types of Gradation	Bitumen Aggregate Ratio (%)	Air Void (%)
Gradation A	8.2	3.1
Gradation B	8.2	4.8
Gradation C	8.2	5.2
Requirements	-	4.5–6.5

**Table 12:** Volume indexes of gradation B.

Bitumen Aggregate Ratio (%)	Air Void (%)	VMA (%)	VFA (%)
8.2	5.2	21.59	77.77
Requirements	5.5 ± 1	≥19	—

**Table 13:** Construction temperatures of the CRMB mixture.

Filler–Bitumen Ratio	0.10	0.25	0.40	0.50	0.60	0.80
Mixing temperature (°C)	178–185	181–188	183–189	185–192	188–195	189–196
Compaction temperature (°C)	162–169	165–172	167–174	169–180	171–178	171–179
